# Evaluation of Functional Electrical Stimulation to Assist Cycling in Four Adolescents with Spastic Cerebral Palsy

**DOI:** 10.1155/2012/504387

**Published:** 2012-05-22

**Authors:** Ann Tokay Harrington, Calum G. A. McRae, Samuel C. K. Lee

**Affiliations:** ^1^Interdisciplinary Program in Biomechanics and Movement Science, University of Delaware, Newark, DE 19716, USA; ^2^Biomechanics Division, ARCCA Inc., Penns Park, PA 18943, USA; ^3^Department of Physical Therapy, University of Delaware, Newark, 301 McKinly Laboratory, DE 19716, USA; ^4^Research Department, Shriners Hospital for Children, Philadelphia, PA 19140, USA

## Abstract

*Introduction*. Adolescents with cerebral palsy (CP) often have difficulty participating in exercise at intensities necessary to improve cardiovascular fitness. Functional electrical stimulation- (FES-) assisted cycling is proposed as a form of exercise for adolescents with CP. The aims of this paper were to adapt methods and assess the feasibility of applying FES cycling technology in adolescents with CP, determine methods of performing cycling tests in adolescents with CP, and evaluate the immediate effects of FES assistance on cycling performance. *Materials/Methods*. Four participants (12–14 years old; GMFCS levels III-IV) participated in a case-based pilot study of FES-assisted cycling in which bilateral quadriceps muscles were activated using surface electrodes. Cycling cadence, power output, and heart rate were collected. *Results*. FES-assisted cycling was well tolerated (*n* = 4) and cases are presented demonstrating increased cadence (2–43 rpm), power output (19–70%), and heart rates (4-5%) and decreased variability (8–13%) in cycling performance when FES was applied, compared to volitional cycling without FES assistance. Some participants (*n* = 2) required the use of an auxiliary hub motor for assistance. *Conclusions*. FES-assisted cycling is feasible for individuals with CP and may lead to *immediate* improvements in cycling performance. Future work will examine the potential for long-term fitness gains using this intervention.

## 1. Introduction

Cerebral Palsy (CP) is a nonprogressive disorder that results from a disturbance in the fetal or infant brain [1, 2]. This disturbance, although varied in etiology, results in motor impairments in the developing child [3–6]. Individuals with CP have muscle weakness and abnormally high muscle spasticity in the affected extremities, which result in fine and gross motor developmental delays [2, 4, 5, 7]. Poor selective muscle control often results in coactivation of agonist and antagonist muscle groups [5]. Spasticity and abnormal tone that is present in the muscles of children with CP [7, 8] can cause abnormal forces at the joints, which can lead to bony deformity, joint instability, and muscle contractures as the child grows [7, 9–11]. The weakness that affects these muscles results in balance impairments and poor selective motor control which may lead to diminished independence and a lack of physical activity [12, 13]. Although CP is a nonprogressive injury of the brain, the impairments and functional limitations associated with CP can change over time, with many children becoming less independent with functional mobility as they enter their teenage years [14, 15]. Traditional means of exercise, such as running, jumping, and playing organized sports, may be difficult for individuals with such functional limitations and, unfortunately, many individuals with disabilities participate in less physical activity than people without disabilities [16–19].

Stationary recumbent cycling has been proposed as a feasible method to exercise in this population [20–29] because cycling does not require the dynamic balance that exercise in a standing position requires. Cycling provides a potential means of improving strength, cardiovascular function, and range of motion, while exercising in a safe position and performing an activity that most children enjoy [26, 27, 29, 30]. Unfortunately, children with CP may not have the strength or coordination to cycle at the power intensities or sustained intervals required to achieve cardiovascular benefits from exercise [21, 31]. Many children with CP have difficulty with the motor performance of the cycling task because of unsmooth, asymmetrical cycling resulting from uncoordinated pushing and pulling on the pedals rather than cycling in a continuous manner [20, 21, 28]. Further difficulties include agonist/antagonist muscle coactivation, poor gross mechanical efficiency with the cycling task, and difficulties attaining threshold heart rates and cycling intensities necessary to achieve cardiorespiratory training effects and musculoskeletal changes [31]. Thus, additional means may be necessary to improve cycling ability for fitness attainment in children with CP.

Functional electrical stimulation (FES) has been used to facilitate cycling to improve cardiorespiratory fitness and to cause musculoskeletal gains in individuals with complete paralysis due to spinal cord injuries (SCI) [32–38]. In addition to cardiorespiratory and musculoskeletal gains, Krause et al. found that FES cycling may also moderate the excessive muscle tone that is present in individuals with SCI [39]. Children with spastic CP have lower levels of cardiovascular fitness [12], muscle weakness [3–5, 7, 8, 40], and elevated muscle tone [7, 11] that also could potentially respond to a FES cycling intervention [24, 41, 42]. The application of FES in individuals with CP, however, is fundamentally a different task than the application of FES in individuals with complete SCI because individuals with CP have varying degrees of volitional ability to pedal a cycle and additionally have sensate lower extremities. Preliminary work in our laboratory [24, 25] examined the development of a FES system for assisting and evaluating cycling in individuals with CP. Two recent publications have also reported the application of FES in individuals with CP [41, 42]. The first publication was a case study featuring training with FES cycling in an adult with CP [41]. The second publication reported on the use of FES in children with CP [42]; however, unlike in the current project, the participants were asked to passively allow FES to propel the crank rather than using volitional effort to contribute to the cycling task. The goal of FES assistance in the present study is to increase the cadence and power output that can be produced volitionally during cycling such that adolescents with CP can reach the heart rate thresholds necessary to gain cardiovascular benefits from exercise. There is also the potential that increasing cardiovascular fitness and strength may lead to improved function and quality of life. The aims of this study were to (1) adapt methods and assess the feasibility of applying FES cycling technology in adolescents with CP, (2) determine methods of performing cycling tests in adolescents with CP of GMFCS levels III-IV, and (3) Evaluate the *immediate* effects of FES assistance on cycling performance (i.e., cycling cadence and power output, coefficient of variance of cycling performance measures, and cardiovascular responses). Preliminary results have been presented elsewhere [25].

## 2. Materials and Methods

### 2.1. Participants

 Participants were recruited from the Cerebral Palsy Clinic at Shriners Hospital for Children, Philadelphia, or through referral from community physical therapists. Adolescents with CP of Gross Motor Function Classification System (GMFCS) levels III-IV were recruited for this study [43]. Participants of GMFCS levels III-IV were targeted because these individuals have the physical capacity to learn how to cycle but they are often limited in their physical activity opportunities due to their lower level of independence with physical mobility. Individuals of GMFCS level III are able to ambulate with the use of an assistive device but may use a wheelchair for long distances. Individuals of GMFCS level IV have limited self-mobility and may require power mobility in the community. The participant inclusion and exclusion criteria are summarized in Table 1.

All participants were screened by an orthopedic surgeon to ensure that they were not at risk for hip dislocation and by a physical therapist to verify that they had sufficient passive range of motion to complete a revolution of the crank comfortably. The informed consent document, including all accompanying procedures and risks, was discussed with each participant and his/her parent or guardian. Sufficient time was provided for review of the document and to answer any questions. Informed consent and assent documents, approved by the governing Institutional Review Board, were signed by each participant and their parent or guardian.

 A sample of convenience consisting of four participants (2 male) with spastic CP between the ages of 12–14 years (mean 13 ± 1.2 years) participated in this case-based pilot study (demographic information is provided in Table 2). Participant 1 had previous cycling experience in the community using an upright tricycle and had previously used neuromuscular electrical stimulation in an isometric strengthening protocol. Cycling and the application of surface electrical stimulation were novel to participants 2, 3, and 4.

### 2.2. Equipment

**Figure 1:**
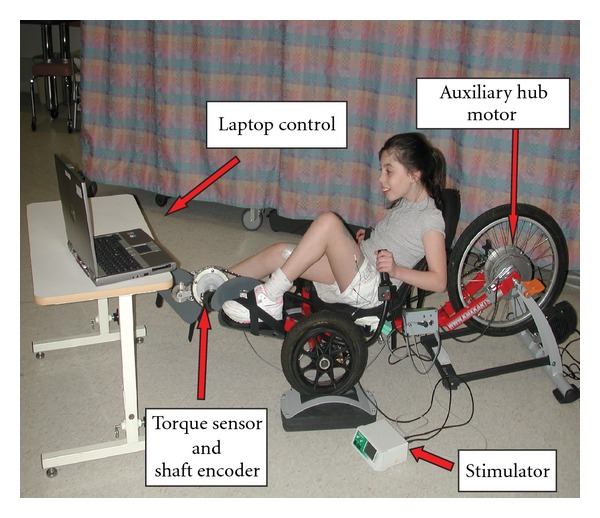
Tricycle components and participant set-up for FES-assisted cycling study [24]. The tricycle-based system is instrumented with a torque sensor and shaft encoder to allow for collection of torque, crank position and cadence, and consequentially the calculation of instantaneous power output, during the cycling session. The stimulator provides surface stimulation to bilateral quadriceps. The auxiliary hub motor was used for subjects 3 and 4 (please see text for details). A laptop computer is used for data acquisition, control of the stimulation timing, and control of the hub motor and to provide visual feedback on cycling performance to the cyclist.

A previously described custom recumbent tricycle-based FES system was used for all testing sessions [24]. This tricycle-based system (a sport tricycle (KMXKarts; United Kingdom) mounted on a cycle trainer (Tacx; Wassenaar, The Netherlands) to allow for stationary cycling) was instrumented with a torque sensor and shaft encoder to allow for collection of torque, crank position and cadence, and consequently the calculation of power output during the cycling session (Figure 1). The tricycle-based system also allows for optional, direct drive pedaling assistance by an auxiliary hub motor directly coupled to the rear wheel's fixed gear. The use of this motor allows for controlled mechanical propulsion assistance in addition to the muscle contraction assistance provided by FES. Data were collected using custom software (MatLab, The Mathworks, Inc). The seat position, crank arm length, bottom bracket, and foot pedal position were adjusted to accommodate the leg length of each participant and any soft tissue contractures. In addition, shank guides previously used in our laboratory [22–24] were used to prevent excessive hip abduction and adduction during the cycling motion.

For this investigation, only the bilateral quadriceps femoris muscles were stimulated during the limb extension phase of the cycling crank rotation using a commercially-available stimulator with custom programming (Hasomed RehaStim, Magdeburg, Germany). Self-adhesive electrode sizes were selected for each individual to maximize the surface area over the quadriceps being activated in an effort to minimize stimulation current density and thereby maximize participant comfort. The appropriate electrode size for all participants was 7.5 × 10 cm (Axelgaard Manufacturing Co., Fall Brook, CA). The proximal electrode was positioned in an oblique orientation over the proximal rectus femoris and vastus lateralis heads of the quadriceps femoris and the distal electrode was positioned in an oblique orientation over the vastus medialis obliquus and distal vastus lateralis heads of the quadriceps femoris. Care was taken to ensure that electrodes were placed over an area of the skin free from blemishes or skin breakdown.

### 2.3. Stimulation Levels

The participants in this study completed training at a stimulation frequency of 33 Hz based on the parameters used in our laboratory for FES cycling in individuals with SCI [24, 35, 44]. A current intensity of 40 mA was well tolerated and allowed for easy facilitation of a fused quadriceps contraction using the electrical stimulation [24]. The level of stimulation applied was then varied by using a throttle (potentiometer) to increase or decrease the stimulus pulse width to control the strength of elicited muscle contractions. A custom program (MatLab, The MathWorks, Inc) controlled the on and off time for the stimulation applied to each leg based upon the position of the crank and the instantaneous cadence at which the participant was cycling [24]. Participants were instructed to tell investigators to turn down or turn off the stimulation at any time if they felt uncomfortable. Participants were also provided with a kill switch that could be pressed to terminate the stimulation at any time during the testing procedures.

Motor level stimulation, defined as the level of stimulation required to cause a muscle contraction that moved the lower extremity through a pedaling arc of motion, was used for FES-assisted cycling trials. Participants were positioned on the bike with the leg being stimulated flexed at the knee and hip and the pedal positioned at the crank angle just prior to where active knee extension occurs. Participants were asked to relax their muscles and allow the electrical stimulation to move their legs. The stimulus pulse width was gradually increased until the limb moved through an arc of motion into extension. This procedure to determine the motor level response pulse width was then repeated until three successive trials were consistent (i.e., with motor level response pulse width values within 5% of each other). This procedure for determining motor level pulse width was then repeated for the opposite extremity. The motor level response pulse width for each individual was used for all FES-assisted cycling tests. Pulse width settings ranged from 90 to 200 *μ*sec (160, 100, 90, and 200 *μ*sec for participants 1, 2, 3, and 4, resp.).

### 2.4. Cycling Tests

**Figure 2:**
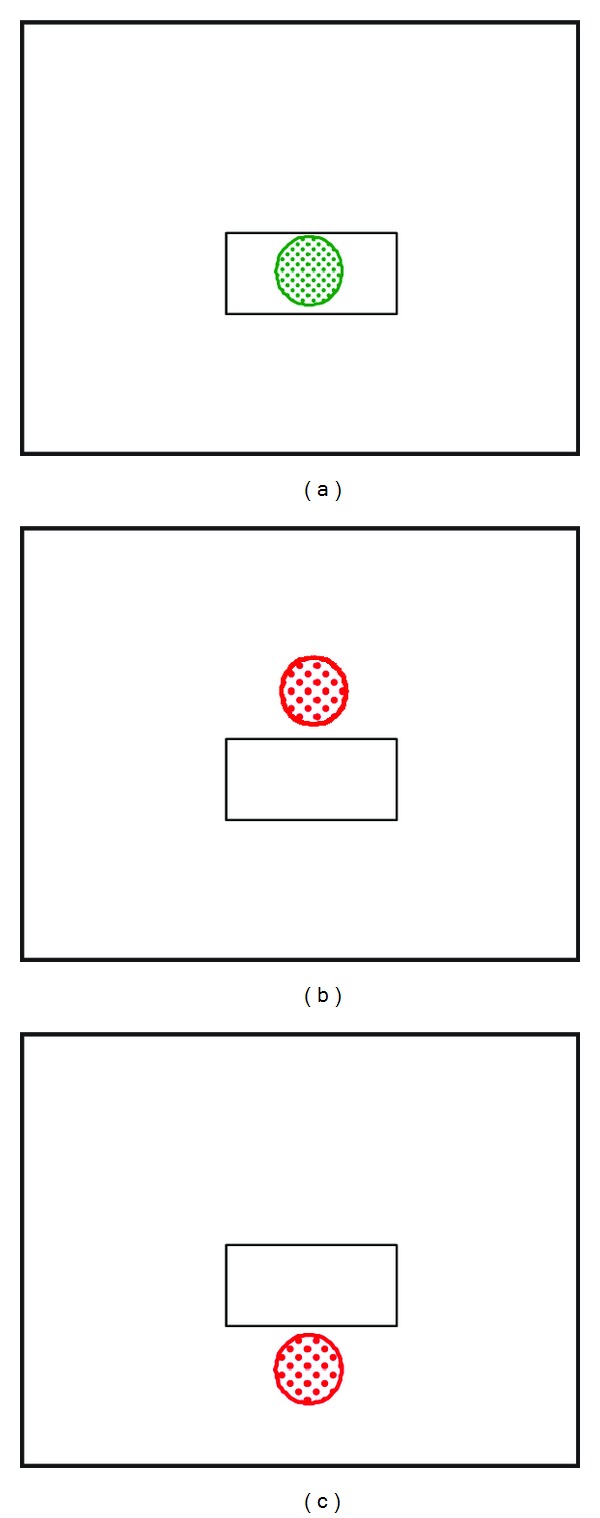
Visual feedback provided during cycling tests and training sessions. Participants are asked to cycle at a target power level or cadence which is represented by the white box on the screen. If the participant is successful, the ball stays within the box and turns green (a). If the participant cycles at a higher (b) or lower (c) power level or cadence, the ball moves out of the box and turns red.

To quantify the immediate effects of applying FES assistance, all participants completed cycling trials *with *and* without* the application of FES assistance. As part of the development of testing procedures, custom software was used to provide a simple feedback system for all participants. A laptop computer provided participants visual feedback of cycling performance with either power output or cadence targets to sustain (Figure 2).

Feedback targets were determined for each participant based on their cycling ability. Some individuals' cycling sessions were essential to the development of cycling performance testing procedures (e.g., use of hub motor to maintain a minimum cadence, discussed hereinafter), while the data from other individuals were used to determine the FES cycling techniques (e.g., application of alternating periods of stimulation on and off in participant 1). The specific tests and total number of tests each participant completed depended upon the stage of development of the project and aim being addressed when the participant participated and the number of times the participant was able to come to Shriners Hospital for testing (Table 3). Participants completed 2–4 sessions with at least 24 hours of rest between cycling sessions and with all testing occurring within a two-week period for each participant. Heart rate was recorded every 10 seconds, for the duration of the test, using a pulse oximeter. 

#### 2.4.1. Incremental Load Tests (Participants 1 and 4)

 The purpose of the incremental load test was to determine peak power output, cadence, and heart rate for each participant. Participants first completed a brief 30-second cycling trial to determine the appropriate power output or cadence increments. During the incremental load tests, the target was increased by equal increments at one-minute intervals. Tests lasted 8–12 minutes as recommended by traditional incremental exercise testing guidelines [45] and were sufficiently rigorous to determine the participant's peak power output.

#### 2.4.2. Constant Load Tests (All Participants)

The purpose of the constant load testing was to assess submaximal performance and determine the participant's ability to work at a steady-state level. For constant load trials, a single power output or cadence target was selected and the participant attempted to maintain the target level for the duration of the test. Constant load tests lasted 8–10 minutes and were sufficient in length for each participant to achieve steady-state cycling performance. For participants completing incremental testing prior to constant load testing, the target for the constant load test was set at 80% of the peak power output achieved during the incremental test. The target for the constant load test was based upon the method of Hunt et al. [46] who examined the energetics of FES cycling in individuals with paraplegia in which the work rate was based on the individual's maximal power on a prior test. Unpublished pilot work in our laboratory determined that exercising at 80% of the incremental load peak power output was sufficient to achieve steady-state oxygen uptake prior to lower extremity fatigue. For participants 2 and 3, who did not complete an incremental load test, the target for the constant load test was set at 80% of the peak power output achieved on the brief, maximal effort, 30-second cycling trial. At least 24 hours of rest was provided between incremental and constant load cycling tests.

#### 2.4.3. Auxiliary Hub Motor (Participants 3 and 4)

 Initial analysis of the first three participants determined that not all participants were able to complete a standard constant load or incremental test without the use of a motor to assist with propulsion. Participants 3 and 4 were unable to cycle consistently enough to maintain target cadences during constant load or incremental load tests. Consequently, the auxiliary hub motor located within the rear wheel of the cycle (Figure 1) was used during some of the testing for participants 3 and 4. The motor facilitated automatic control of cadence while simultaneously collecting power output data to provide information on cycling performance [24, 25]. Because of the poor cycling ability of these participants, the majority of the torque produced was resistive to forward motion of the crank which resulted in a negative net work rate. For these participants, in a period of passive cycling in which the participants allowed the motor to move their legs, negative power output data were collected. This represented how much the individual was resisting the passive movement of their legs and, in effect, how much work the motor had to do to overcome the weight and muscle tone in the participant's legs to turn the crank. After a period of passive cycling, the participants were asked to actively cycle and the difference between the passive phase and active phase power outputs was calculated to determine the net power output for the cyclist. Because cadence was controlled by the auxiliary hub motor for participants 3 and 4, power output targets were used for visual feedback during the cycling tests.

### 2.5. Data Analysis

Cadence and torque data were collected at 20 Hz using custom software (MatLab, The MathWorks, Inc) and used to calculate instantaneous power output. For participants completing constant load tests with and without FES assistance, paired  *t*-tests were used to analyze differences between mean power output between tests with and without FES. Cycling performance was also analyzed by calculating the coefficient of variance of power output for each minute of the active cycling phase and then averaging across the total number of minutes to determine the coefficient of variance for the test. For participants completing the test using cadence as feedback (participants 1 and 2), the coefficient of variance of cadence was also calculated using the standard deviation of cadence and mean cadence in the calculation instead of power output. Paired  *t*-tests were performed to compare coefficient of variance between testing conditions with and without FES assistance.

Peak heart rate during incremental load testing was determined as the maximum of all heart rate data collected in a testing session. Peak heart rate was reported as a percentage above resting heart rate to account for slight within-participant variations in resting heart rate occurring between testing days. Average heart rate reflects an average of all heart rate data collected during the active cycling phase of testing within a single testing session.

## 3. Results

### 3.1. Application of FES Assistance

All participants were able to complete tests without difficulty and were able to tolerate the application of FES to bilateral quadriceps muscles. All participants were able to complete motor level stimulation thresholding and FES-assisted cycling trials without requiring additional acclimation to tolerate the stimulation. Additionally, although participants were provided with the kill switch, none of the participants chose to terminate the stimulation when FES was applied. The stimulus pulse width required to elicit a motor level response ranged from 90 to 200 *μ*sec (see Section 2.3 for participant-specific pulse widths).

### 3.2. Cycling Performance

Participants 1 and 2 completed cycling tests *without* the use of the auxiliary hub motor to maintain a constant cadence. Cadence was used as a target for the constant load and incremental tests in these participants. Participant 1 completed two separate incremental tests (with and without FES assistance) and a constant load test in which FES assistance alternated between on and off at one-minute increments over the length of the test. Participant 2 completed constant load trials with and without FES assistance.

 Participants 3 and 4 completed cycling tests *with* the auxiliary hub motor assistance. Power was used as the feedback target for the constant load and incremental tests for these participants. Baseline passive cycling data were collected to determine the amount of work the motor needed to perform to passively move the legs through a range of motion without the participant assisting with the task. Participant 3 completed three constant load tests: one *without* the use of the auxiliary hub motor and *without* FES assistance, one *with* the use of the auxiliary hub motor and *without* FES assistance, and one test* with* both the auxiliary hub motor and FES assistance. Participant 4 completed brief cycling trials with and without the use of the auxiliary hub motor and she completed three tests *with* the use of the auxiliary hub motor: an incremental test *without* FES assistance and constant load tests *with *and *without* FES assistance.

#### 3.2.1. Incremental Load Test Results (Participants 1 and 4)

**Figure 3:**
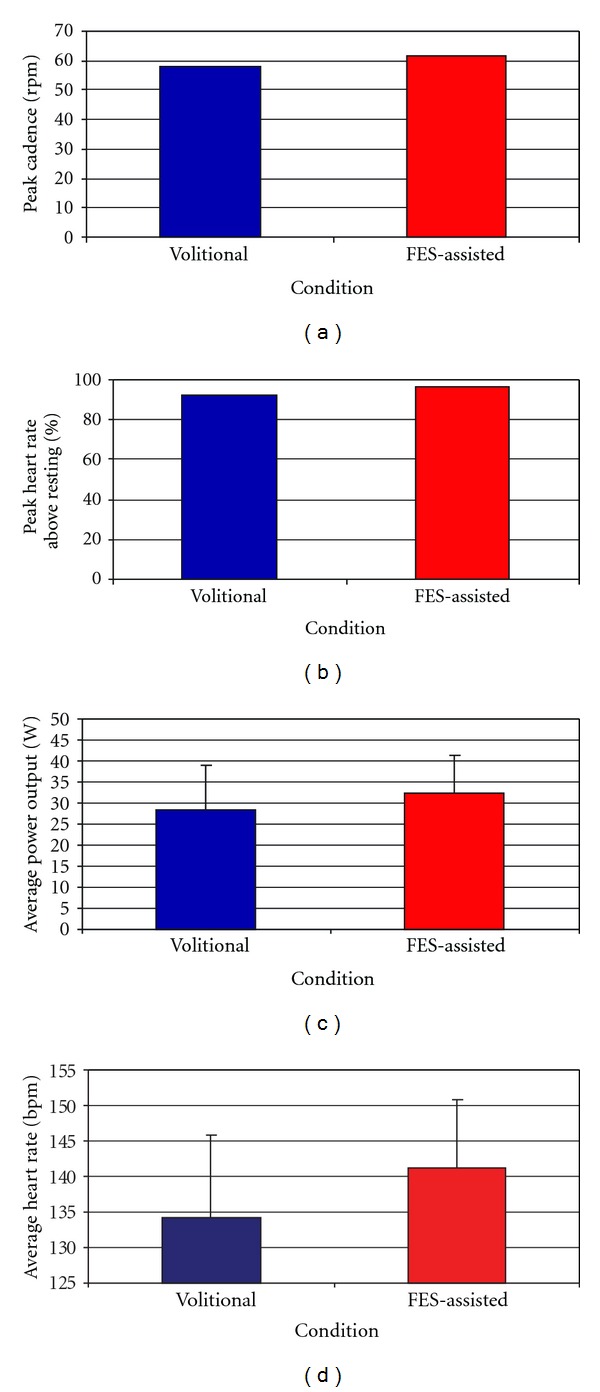
Cycling performance during incremental cycling tests with and without FES assistance in a child who is well adept at cycling (participant 1). The graphs illustrate peak cadence (a), peak heart rate as a percentage of resting heart rate (b), average power output (c), and average heart rate (d) during the tests. For average power output (c) and average heart rate (d) standard deviation values are shown. The blue bars represent volitional cycling and the red bars represent FES-Assisted cycling trials.

 Only participant 1 completed incremental load tests with and without FES assistance. During incremental cycling tests, this participant achieved higher peak cadence and heart rate values during the test with FES assistance (Figures 3(a) and 3(b)). The primary objective of the incremental test was to determine peak heart rate and power output values; however, a secondary analysis examined average heart rate and power output across the tests to determine the relative level of exertion at which the participant was working over the duration of the test (Figures 3(c) and 3(d)).

Cycling performance was also analyzed by calculating the coefficient of variance (averaged over each minute during the cycling trial) in power output and cadence. Participant 1 demonstrated a decrease in the variability in cadence (mean 16.3 ± 4.3% without FES assistance versus 7.8 ± 2.1% with FES assistance) and power output (mean of 28.8 ± 4.5% without FES assistance versus 16.5 ± 2.5% with FES assistance) in the cycling test with FES applied.

The incremental load test completed by participant 4 was performed as part of development of incremental load testing procedures using the auxiliary hub motor. The participant was able to complete the testing and appropriately respond to the increasing target on the computer screen. He did not complete a FES-assisted incremental load test that could be used for comparison.

#### 3.2.2. Constant Load Test Results (All Participants)

**Figure 4:**
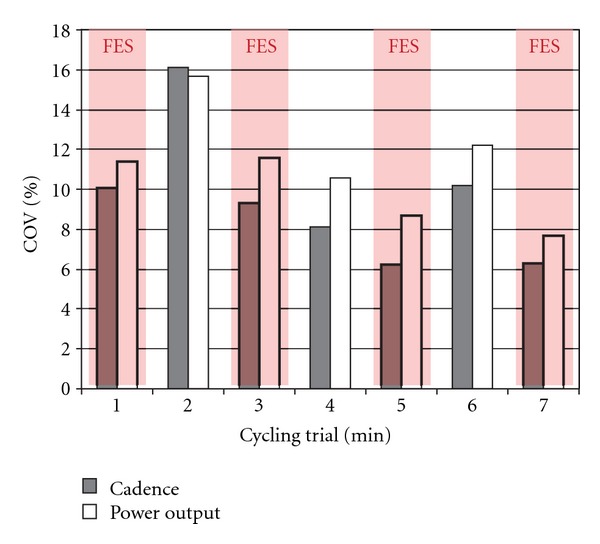
Coefficient of variance of cycling cadence and power output during a constant load cycling test in an individual with CP who is adept at cycling (Participant 1). The coefficient of variance for each variable was calculated over 1-minute periods in which FES assistance to the quadriceps muscles was either turned on (red shaded areas) or off (areas of the graph without red shading).

Participant 1 completed a constant load test in which FES assistance was alternated in one-minute increments of being on and off. The coefficient of variance for this participant's power output and cadence were calculated across each minute. Overall, the participant demonstrated an immediate decrease in the variability in cadence (mean 11.5 ± 4.1% without FES assistance versus 8 ± 2% with FES assistance) and variability of power output (mean 12.8 ± 2.6% without FES assistance versus 9.9 ± 2% with FES assistance) during the periods when FES assistance was applied (all *P* values > 0.3) (Figure 4). Although changes in variability were not statistically significant, it did appear that participant 1 cycled more smoothly with FES.

**Figure 5:**
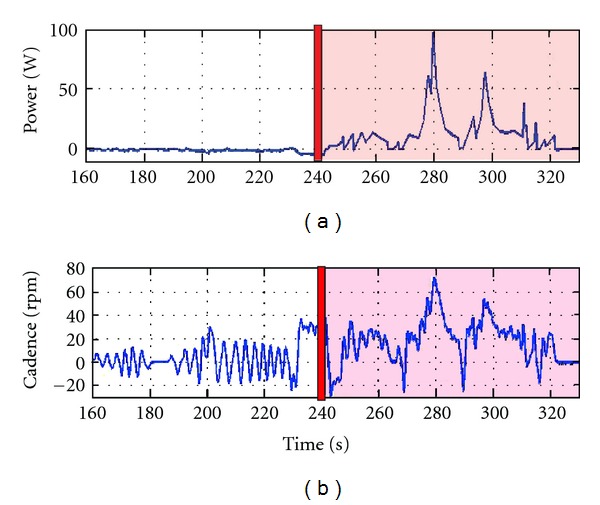
Cycling performance during the initial application of FES in a child for whom cycling was a novel task (participant 2). The top trace (a) illustrates his power output and the bottom trace (b) illustrates his cadence. The red vertical bars at 240 s indicate when FES assistance began and FES remained on during the red-shaded portion of the graph. Data were smoothed for analysis using a second-order lowpass Butterworth filter with a cutoff frequency of 0.1 Hz.

The application of FES assistance led to immediate changes in cycling performance for participant 2, who had no cycling experience and he was unable to complete a crank revolution without assistance. As described previously, Participant 2 completed constant load trials (full tests were unable to be completed due to poor cycling ability and this participant participating prior to the implementation of the auxiliary hub motor) with and without FES assistance. During volitional trials without FES assistance, his lower extremities would get stuck during his attempt to pedal forward and he would alternate between pedaling approximately 180 degrees forward and backward. The forward and backward motion of the crank resulted in no net power generation (Figure 5(a): prior to the application of FES assistance) and a cadence that fluctuated from positive to negative (Figure 5(b): prior to the application of FES assistance). Once FES assistance was applied, the participant was able to successfully pedal the cycle (Figure 5).

**Figure 6:**
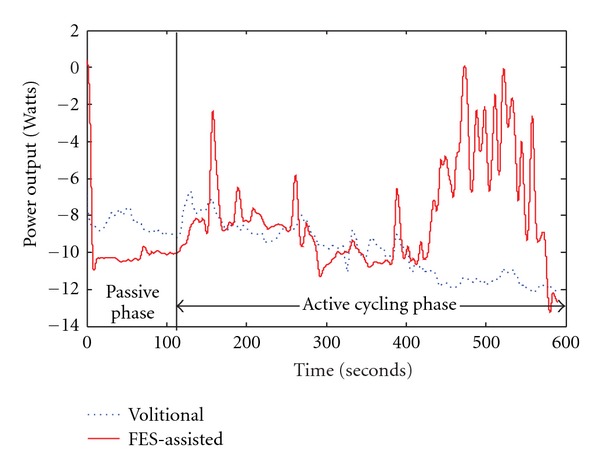
Power output during constant load cycling tests with (red line) and without (blue line) FES assistance. This data are from a child without cycling experience (participant 3) and the auxiliary hub motor was required for testing. The vertical black line at 120 seconds denotes the transition from a passive cycling phase (in which the motor moved the child's legs while he rested) to an active phase (in which the motor continued to control cadence, and the participant assisted with the cycling effort). In the FES assistance condition, the stimulation was applied only during the active cycling phase. Data were smoothed for analysis using a second-order lowpass Butterworth filter with a cutoff frequency of 0.1 Hz.

Participant 3 required the use of the hub motor to perform constant load cycling tests. During the volitional cycling test, the participant's power output was negative throughout the majority of the cycling task, although positive power output was achieved during portions of the FES-assisted constant load test (Figure 6). Participant 4 also completed constant load trials with and without FES assistance; paired samples  *t*-tests comparing average power output during two constant load cycling test conditions (with and without FES assistance) for participants 3 and 4 were not statistically significant (*P* = 0.846).

## 4. Discussion

FES-assisted cycling was well tolerated by the participants in this study and demonstrated the feasibility of applying FES assistance in children with CP. The use of an auxiliary motor to control cadence was necessary to allow for meaningful assessment of cycling performance in some participants. FES-assisted cycling resulted in increased cadence, power output, and heart rates and decreased variability in cycling performance compared with volitional cycling without FES assistance. Such improvements in cycling ability with the application of FES assistance may make cycling for fitness attainable for individuals with CP who have impaired cycling ability.

### 4.1. Application of FES Assistance

All participants were able to tolerate the application of FES and complete testing. Only one participant (participant 1) had previous experience with surface electrical stimulation while the other three did not. Unlike in individuals with complete SCI who are unable to volitionally contribute to the cycling task, our participants were able to cycle volitionally, although inefficiently, and were provided with visual feedback on the computer screen to encourage cycling at a target cadence or power output (Figure 2). The use of this feedback system may also have applications in studies involving children with incomplete SCI or poststroke because these individuals may have some ability to contribute to the cycling task [47]. In a recent study by Trevisi et al., participants with CP were asked to not contribute to the cycling effort and to allow the cycle to move their legs during the passive cycling and FES cycling phases [42]. Although the authors did find that FES in conjunction with traditional rehabilitation led to greater improvements in quadriceps strength and motor control than traditional rehabilitation alone, these gains may have been even greater had the participants trained using volitional effort combined with FES assistance. In addition, unlike the stationary ergometers used in previously published studies on FES cycling in individuals with CP [41, 42], the tricycle-based system in this study is capable of overground applications [24].

### 4.2. The Use of an Auxiliary Hub Motor (Participants 3 and 4)

This work demonstrated that not all children with CP are able to pedal a tricycle without assistance. The use of the auxiliary hub motor allowed us to run quantitative exercise tests at clearly defined power increments. The work of Johnston et al. and our own pilot work have demonstrated the erratic cycling patterns used by children with CP of GMFCS levels III and IV [21, 24]. By using the auxiliary hub motor, we were able to quantify cycling performance in individuals with poor cycling ability. This information is difficult or impossible to gather without the use of the motor assistance due to the low, erratic, or negative work rates. For example, participant 3 was unable to cycle at a target power output of 0 Watts without motor assistance. Thus, he did not have the strength and coordination to volitionally cycle while working against only the resistance of his own legs. This resistance can be thought of as retarding torque which can be caused by muscle coactivation and muscle spasm, friction from the cycle drive train, and inertial resistance from the mass of the limbs. The cycling system, motor, and software, however, allowed us to collect power output data, despite the fact that the power output was less than 0 W. We discovered that during passive cycling, with the motor propelling the individual's legs, the power recorded was −8 W, indicating that the motor assistance required to passively move his limbs through the revolution was 8 W (Figure 6). With the use of the auxiliary motor and software, we were able to determine that the individual can contribute approximately 4 W of cycling effort for a net power output of −4 W. The contributing effort of the individual would be immeasurable with a nonmotorized system because of the inability to record negative power output for the individual unable to complete a crank revolution without motor assistance.

### 4.3. Cycling Performance

Using FES-assisted cycling technology in adolescents with CP is a novel translational approach from the treatment of individuals with SCI [24, 25]. The results demonstrated that FES-assisted cycling can be used to facilitate cycling in children who cannot cycle independently (Figure 5). Although in some cases the application of FES produced immediate increases in power output (Figure 5(a)), in others it took several minutes to see a significant increase in power (Figure 6). In the participant who required a few minutes of FES assistance to demonstrate increased power output (Figure 6), however, the early phase of active FES-assisted cycling also demonstrated a decrease in his resistive torque (he was fighting the passive movement of his legs less than in the volitional test).

#### 4.3.1. Incremental Load Tests

The application of FES assistance led to increased peak power output and heart rate during incremental cycling tests (Figures 3(a) and 3(b)). This participant also maintained a higher average heart rate when cycling with FES assistance (Figure 3(d)). The data from this participant demonstrates that FES assistance can facilitate an elevated heart rate while cycling. Thus, we hypothesize that FES-assisted cycling training has the potential to allow children with CP to cycle more vigorously and achieve therapeutic levels of effort which could result in improved cardiovascular fitness in individuals with CP who are not able to exercise in a traditional manner [12, 16–18].

Additionally, FES assistance also decreased the variability in cycling cadence and power output. Decreased variability in cadence signifies improved cycling performance and better potential for carryover into prolonged endurance cycling and overground cycling applications.

#### 4.3.2. Constant Load Tests

 Participants performing constant load tests also demonstrated increased power output and decreased variability in cadence and power output, which were immediate, when FES assistance was applied (Figures 4 and 6). In participant 3, the power output recorded in the volitional effort test (Figure 6—blue trace) decreases following the passive cycling phase; thus the motor had to work harder to turn the crank when the individual was helping than it did when he was resting and allowed the motor to move his legs passively. This observation demonstrates participant 3's inefficiency and unproductive volitional cycling ability, and we hypothesize that this is due to the increased coactivation and mechanical inefficiency of cycling which results in a lower net torque and an increase in the resistive torque. The decrease in power output observed in the volitional test (Figure 6—blue trace) was not present when FES assistance was applied to assist with muscle contraction timing (Figure 6—red trace). This participant was able to use less resistive torque when the FES assistance was applied. We hypothesize that with further training and FES assistance, this participant may have the potential to begin to produce positive torque and power output when cycling with the use of the auxiliary hub motor. Although the differences between FES-assisted and volitional cycling conditions were not statistically different, we anticipate that changes would be present with a larger sample size.

### 4.4. Limitations

 The conclusions on the application of FES assistance on cycling in children with CP are limited due to the small sample size and the pilot nature of this preliminary work. The investigators acknowledge the risk of selection bias because (1) the participants were not randomly selected, (2) participants were required to have sufficient cognition and communication skills to participate in testing and training procedures, and (3) patients and families volunteering to participate may not be representative of the general population of children with CP. In addition, due to participant availability, not all participants completed a full series of constant load and incremental tests with and without FES assistance. This work was also limited in that metabolic data were not captured to provide additional insight on oxygen consumption and metabolic efficiency during cycling tests with and without FES assistance.

## 5. Conclusions

The objectives of this study were to (1) adapt methods and assess the feasibility of applying FES cycling technology in adolescents with CP, (2) determine methods of performing cycling tests in adolescents with CP of GMFCS levels III-IV, and (3) evaluate the *immediate *effects of FES assistance on cycling performance. Functional electrical stimulation-assisted cycling was tolerated well and resulted in increased cadence, power output, and heart rates and decreased variability in cycling performance. Such improvements in cycling ability with the application of FES assistance may make cycling for fitness attainable for individuals with CP with impaired cycling ability. The use of an auxiliary motor to control cadence may be necessary to perform quantitative cycling tests in individuals with CP with poor cycling ability. This study was designed to evaluate FES-assisted cycling as a method of improving cycling ability and to develop evaluation methods for use in future studies designed to assess potential benefits of this type of exercise intervention. Novel techniques such as FES-assisted cycling have the potential to provide a method for exercise and fitness for individuals with CP whose physical impairments limit their level of physical activity. With FES-assisted training and the immediate improvement in cycling ability it produces, there is the potential for carryover into overground cycling to provide recreational opportunities for individuals with CP. Future work will focus on determining the optimum stimulation settings for this approach and an evaluation of the effect of FES-assisted cycling training on cycling and cardiovascular performance.

## Figures and Tables

**Table 1:** Inclusion and exclusion criteria for participation.

Inclusion	Exclusion
(i) 10–18 years of age	(i) Diagnosis of athetoid or ataxic CP
(ii) Diagnosis of spastic diplegic or quadriplegic CP	(ii) Significant scoliosis with primary curve >40 degrees
(iii) GMFCS level III (walks with assistive device; may use a wheelchair for long distances) or IV (self-mobility with limitations; transported or uses power mobility in the community)	(iii) Spinal fusion extending into the pelvis
(iv) Sufficient covering of the femoral head in the acetabulum (MIGR% < 40%)	(iv) Severe tactile hypersensitivity
(v) Adequate range of motion of the hips, knees, and ankles to allow pedaling	(v) Joint instability or dislocation in the lower extremities
(vi) Sufficient visuoperceptual skills and cognition/communication skills to participate in cycling trials	(vi) Surgery, traumatic or stress fractures in the last year
(vii) Seizure-free or well-controlled seizures	(vii) Botulinum toxin injections in the LE muscles in the past 6 months
(viii) Willingness to participate in testing at Shriners Hospital for Children, Philadelphia	(viii) Severe spasticity of the leg muscles (e.g., a score of >4 on the Modified Ashworth Scale)
(ix) Ability to communicate pain or discomfort with testing procedures	(ix) Joint pain or discomfort during cycling
(x) Ability to obtain parent/guardian consent and child assent/consent	(x) Severely limited range of motion/irreversible muscle contractures that prevent the subject from being able to be safely positioned on the cycling
	(xi) History of pulmonary disease limiting exercise tolerance (Asthma Control Test screen) or history of known cardiac disease (American Heart Association Screen)
	(xii) Pregnancy

**Table 2:** Participant description and FES parameters for cycling tests with FES.

Participant	Gender	Age (years)	Type of spastic CP	GMFCS level	Mode of community mobility
1	F	12	Diplegic	III	Anterior rolling walker
2	M	14	Diplegic	III	Posterior rolling walker
3	M	14	Quadriplegic	III	Posterior rolling walker; manual wheelchair at school
4	F	12	Quadriplegic	IV	Manual wheelchair

GMFCS refers to gross motor function classification scale level [43].

**Table 3:** Overview of Tests Completed by Each Participant.

Participant	No motor constant load VOL	No motor constant load FES	No motor incremental load VOL	No motor incremental load FES	Motor constant load VOL	Motor constant load FES	Motor incremental load VOL	Other cycling trials completed
1			**×**	**× **				No motor constant load with FES alternating on:off in 1 minute increments

2	**×**	**×**						

3	**×**				**×**	**×**		

4					**×**	**×**	**×**	No motor-Brief trial without FES assistance

Participants are listed by row with “**×**” corresponding to each test a given subject completed. “No motor” refers to cycling tests without the use of the auxiliary hub motor to control cadence, while the hub motor was used in the “Motor” tests. VOL refers to tests without the use of FES assistance. FES refers to tests in which FES was applied. For the tests in which FES was applied, stimulation was applied at 33 Hz, 40 mA. Pulse width ranged from 90 to 200 *μ*sec and corresponded to the participant-specific pulse width required to elicit a motor level contraction. Participants 1 and 2 used cadence as feedback on cycling performance, while participants 3 and 4 used power output for feedback due to requiring the use of the auxiliary hub motor (please see text for details).
